# Case of Gun-Shot Wound of the Shoulder Joint, with Lodgement of the Ball in the Head of the Humerus, Fracture of the Scapula, &c. Wherein Amputation at the Shoulder Joint, under Unfavourable Circumstances, Was Successfully Performed

**Published:** 1818-07-01

**Authors:** William Burnett

**Affiliations:** Chichester


*Case of Gun-shot Wound of the Shoulder Joint, with Lodgement of the Ball in the Head of the Humerus, Fracture of the Scapula, &c. wherein Amputation at the Shoulder Joint, under unfavourable Circumstances, was successfully performed.* By WILLIAM BURNETT, M. D. of Chichester.

Philip Chesse, a French prisoner of war, was wounded at the battle of Corunna, and fifteen days thereafter was sent to Forton hospital. On examination, it was found that a musket ball had passed through a portion of the Trapezius muscle, in the direction of the joint; but whether it was there lodged, I could not at the time ascertain, the whole of the limb (but particularly around the shoulder) being much swoln, and exceedingly painful; and there was a considerable degree of active inflammation in the neighbourhood of the wound, from the orifice of which a little well-formed pus was discharged. The shoulder was wrapped in an emollient cataplasm, he was put to bed, and took an anodyne draught.

On the following day (26th January) he complained of pain in the chest, which, on inspiration, was very acute; his face was flushed, and the pulse full and hurried. He was bled to sixteen ounces, took a purge of neutral salts, and was put on low diet. In the evening his pain was not much lessened, and he spat blood; he also complained of a troublesome cough. Blood was again drawn to the same extent, and an expectorant mixture ordered for his cough.

27th. The pain in the chest is much less, but the cough is still frequent, and he expectorates mucus streaked with blood; pulse small and 120; tumefaction around the shoulder continues to increase, and an obscure sense of fluctuation is felt in several parts; there is very little discharge from the wound. Cataplasm and pectoral mixture continued.

From this day to the 31st, he continued much in the same state, when an incision was made in the most dependent part of the tumour, nearly about the centre of the deltoid muscle, and about half a pint of well-formed pus evacuated with great relief. The cataplasm was continued, and for some days, matter was freely discharged from the opening. A swelling then made its appearance, which, on examination, proved to be another formation of matter under the pectoral muscle of the same side, which was attended with much pain, and increased difficulty of breathing.

This tumour was likewise poulticed, and at the end of the third day a free incision was again made into the most depending part, and a profuse discharge of well-formed pus took place, mixed with irregular portions of cellular membrane. He still continued to complain of pain in the chest, and at times spat blood, for which he took a pectoral mixture during the day, and an opiate at night The original wound discharged a little pus. His diet was ordered to consist chiefly of milk and farinaceous substances.

On the 10th, the discharge still continuing great, and the febrile symptoms much reduced, he was allowed a gill of wine in the day. The wound was this day dressed simply.

15th. The discharge is certainly lessened, but may still be called profuse; the pulse, which during the day is from 75 to 80, at night ranges above 100; the pain in the chest and cough have, however, entirely ceased. He was now put on an increased diet; the wine was continued, and the different wounds dressed twice a-day. From this time the discharge from all parts gradually lessened, the tumefaction ceased, and the incisions healed up; and now the head of the humerus could be distinctly felt, fractured in the glenoid cavity. As he was much debilitated, he was put on full diet, and took decoct. cinchon. c. acid. sulphur. dilut. ter die.

He remained in this state till the beginning of April, when all discharge, except from the original wound, had for some time ceased. In the course of this period, I had various consultations with some of my medical friends, as to the propriety of removing the limb at the articulation; and the general opinion having been unfavourable, I had hitherto deferred the performance of that operation. About the beginning of the month first mentioned, he began to complain of profuse night sweats, and had other well marked symptoms of the commencement of hectic fever; the discharge from the wound had increased of late, and was become thin and fœtid; his appetite was bad, and his strength again declining. It was evident from these symptoms, that the removal of the limb was the only chance for the patient; I therefore proposed it to him, and after a few days consideration, he consented.

I performed the operation on the 11th of April, the first step being to dissect the deltoid muscle, and form a flap thereof; my friend, Mr. Heron (as he had on several other occasions for me), perfectly compressed the artery *above the clavicle,* with his finger alone. On dividing the capsular ligament, &c. and exposing the joint, it was found, as had been anticipated, that a partial anchylosis had taken place, and also, that the processus acromion, and posterior margin of the glenoid cavity were wounded, and the ball itself lodged in the head of the humerus, separating the portion more immediately in contact with the glenoid cavity, in its centre down to the neck, from the general shaft of the bone; and on endeavouring to turn the head of the humerus out of the cavity, in order to pass an amputating knife behind, to complete the removal of the limb, this portion remained attached. The incision being carried from above, down into the axilla, the arm separated and the vessels secured, I proceeded to remove the residue of the humerus, and the fractured and diseased parts which yet remained. The first was readily effected by a common levator, and the fractured portions of the acromion and glenoid cavity were, with equal facility, removed by Hey's head saw. The parts being cleaned and examined, the glenoid cavity presented an unfavourable appearance, being black in several places, and covered with clusters of a jelly-like matter. This last being, in a great measure, removed by the sponge, the flap laid down and dressed with adhesive straps, pledget and bandage, he was put to bed, and given an anodyne draught.

The dressings were removed on the 15th, when it was found, that the whole of the superior part of the flap on each side had healed by the first intention; the discharge was good, and the lower part of the flap looked healthy; there was a trifling exfoliation from the original wound; his night's rest since the operation has been good, but his pulse has continued small and quick, with occasional flushings of the face.

From this time the wound was dressed daily, and continued to amend; a little trouble was experienced, as I have found in several similar cases, from the want of adhesion of the flap to the glenoid cavity, and a consequent gleety discharge took place; but this was soon obviated by the attentive use of a compress, secured in its place, both by adhesive straps and the bandage. The ligatures came away at the fifth and sixth dressings, and his general health went on improving. On the 25th, being in every respect much amended, he was allowed the full diet of the hospital. Next day he was uneasy, and towards the evening he had febrile symptoms; on the following morning, the shoulder was found covered by an erysipelatous inflammation. His diet was again reduced, a brisk cathartic prescribed, and his shoulder covered with several folds of linen, wetted in a solution of the plumbi superacet. to which was added a portion of spt. vini. camphor. In a few days, the inflammation ceased, and he was again ordered decoct. cinchon. c. acid. sulphur. dilut. On the twenty-first day from the operation, the cure was completed, leaving only the line of the flap, as a trace of its having been performed.

*Chichester, March,* 1818.

